# Letter from Prof. Flint, Containing a Brief Notice of Certain Strictures on His Report to the Buffalo Medical Association, on Pneumonia; by Dr. Geo. N. Burwell

**Published:** 1856-02

**Authors:** Austin Flint


### BUFFALO MEDICAL JOURNAL

AND

### MONTHLY REVIEW.

**VOL. 11.** FEBRUARY, 1856. **NO. 9.**

### ORIGINAL COMMUNICATIONS.

ART. I.—*Letter from Prof. Flint, containing a brief notice of certain Strictures on his Report to the Buffalo Medical Association, on Pneumonia; by Dr. Geo. N. Burwell.*

Dr. Hunt,

*Dear Sir:* The Buffalo Medical Journal for Dec., contains a paper by Dr. Geo. N. Burwell, of Buffalo, taking exceptions to "a few of the matters of fact and opinion" presented in the report which, as chairman of a committee of which Dr. B. was a member, I had the honor to read to the Buffalo Medical Association in September last.* So far as matters of opinion are concerned, the paper referred to does not call for a reply. But since Dr. Burwell questions the accuracy of certain positions stated as matters of fact, justice to my medical friends who assigned to me the duty of preparing the report, as well as to myself, seems to require that I should either express due acknowledgments for being corrected, or, on the other hand, reäffirm the statements, should it happen that my critic has shown more zeal than success in his efforts to discover errors in my report.

Proceeding at once, and as briefly as possible, to notice the strictures contained in Dr. Burwell's paper, I will take them up *seriatim*, in the order in which they come.

*Stricture No.* 1. Dr. B. says, "a point in my opinion of the highest importance to a correct understanding of the pathology and treatment of pneumonia has not been referred to in the report. I refer to the fact of the excess of fibrin in the blood so long ago pointed out by Andral and Gavarret."

It is a sufficient reply to this stricture to say that "the diagnostic value of the buffy coat" was one of the subjects assigned to the committee to be specially considered. As chairman of the committee, I design to submit a report on this subject at a future time, as I distinctly stated at the close of my report. In connection with this report to be made hereafter, it will be pertinent to inquire into the "question of fact which has strongly impressed itself" upon the mind of Dr. B., that in typhoid pneumonia the fibrin of the blood is below the normal quantity. In the mean time, perhaps Dr. B. may collect some data bearing on this "question of fact" concerning which, in anticipation, he feels "a strong assurance." It will also be appropriate at that time, to consider how far thrusting a stick into a clot *after* bleeding, in order to ascertain whether, or not, it adheres to the stick with sufficient tenacity to sustain its own weight, be a scientific and exact method of settling, in individual cases, the propriety of taking blood.

*Stricture No.* 2. In my report I state that in pneumonia "the inflammation in the adult, in the great majority of cases, extends over an entire lobe at least." Dr. Burwell declares that his observations would not have led him to this conclusion. The remarks which follow I must take the liberty of reproducing. He says, "In the first place I have almost uniformly seen pneumonia show itself first in a spot on one surface, of one lobe, of one lung, and from this spot as a centre spread *centrifugally*,(?) and in bad cases only inwardly into the substance of the lungs. Indeed, if my observations are correct, the disposition of the disease to overleap the sulci between the lobes, and to extend itself on the periphery of an adjoining lobe, is much greater than its disposition to extend inwardly. It is very rarely indeed that I see an entire lobe affected; and the fact of its occurrence always fills me with the liveliest apprehensions for my patient's safety." Here are some very curious statements, which, if I am not mistaken, must surprise most intelligent readers. He has very rarely seen an entire lobe affected! Whence, then, the significance of the title *lobar* pneumonia? My own observations on this point would of course weigh but little with Dr. B. If he refers to authors he will generally not find an explicit enunciation of the fact which I state, simply because it is a matter of common observation aud commonly understood. On turning to Grisolle, however, (a work to which he afterward refers) in the chapter headed *Marche de la pneumonie aigue* (*Traité pratique de la pneumonie*, page 298) he will find an account of some observations by one who deemed blood-letting so important a therapeutical measure in this disease, that he could only conscientiously withhold it in a very few peculiarly mild cases. Out of 152 patients treated, as Grisolle is careful to remark, more or less energetically, in 20 only did the inflammation remain ilmited nearly or entirely to the parts primarily invaded. In all these cases the diffusion of the disease was noted each day with care. Thus in 132 patients, to quote his language (I take the liberty of translating, and Dr. B. will correct me if it be not correctly rendered) "The inflammation which had began by affecting only a sixth, or a fifth, or a quarter of the pulmonary surface, extended more or less rapidly over a half, or two-thirds, or the whole of the organ." This rule is so general that Grisolle likens the march of the affection to erysipelas. The results of Dr. B.'s observation are so peculiar in this regard that I must be allowed to question their accuracy.

Dr. B. is not less peculiar in having observed the inflammation to overleap the sulci oftener than extend inwardly. On this point Grisolle is equally explicit. He says, "It seemed that the interlobar fissure exerted an effect to retard the march of the disease; for, if three cases are excepted, it was found that in every instance in which the pneumonia invaded the upper and lower lobes, the inflammation attacked each of these portions at different periods, and the interlobar fissure marked a transition from one stage to another."

Grisolle, too, did not find that reluctance in the disease to travel inwardly from the superficies which forms another peculiarity of Dr. Burwell's cases. Having stated that he never found in his autopsies hepatization seated exclusively in the center of a lobe, he says, "it is also infinitely rare to see hepatization invade only the outer surface over a certain space." He adds that he met with this anomaly but in a single instance. It may be said, that Grisolle refers to fatal cases; in reply I ask how is it to be determined during life that inflammation extends only a certain distance from the surface toward the centre of a lobe? I should be very glad to be informed what are the physical signs upon which Dr. Burwell bases an opinion that the inflammation does or does not "spread inwardly into the substance of the lungs." There are means of determining that a central pneumonis is advancing to the surface, but to undertake to guage the depth of an inflammation commencing at the surface, is a refinement in stethoscopy which I confess is new to me, as it will be, I presume, to others who profess to have some knowledge of the subject.

Dr. Burwell is "filled with the liveliest apprehension" for his patient's safety when he sees an entire lobe affected. I am happy in not being inconvenienced by such fears. If the lower lobe be alone affected, and the affection remain thus limited, the danger, in my opinion, is chiefly in the gratuitous fears of the practitioner which are likely to lead him to employ blood-letting or other potent therapeutical remedies, not required, and therefore positively injurious. If the inflammation be limited to the upper lobe, there is much more occasion for apprehension if it be circumscribed than if it be lobar, for in the former case it constitutes very strong evidence of tuberculosis. This fact Dr. Burwell will find not to rest on my statement, if he will take pains to inform himself on the subject.

*Stricture No.* 3. I quote from Dr. Burwell's paper as follows: "There is another statement made in the report which I think needs some qualification. It is that 'double pneumonia, *i. e.*, the inflammation seated in both sides of the chest, is exceedingly rare in the adult.' I know of no observations upon this point but those of M. Grisolle. From an examination of 1430 cases, he says that the inflammation was double in 18 per cent. of them, *nearly one-fifth*, a proportion certainly large enough, I think, to shake one's confidence in the opinion that pneumonia tends intrinsically to recovery."

Now it is charitable to conclude either that Dr. Burwell has not undertaken to read Grisolle's treatise on pneumonia, or that his knowledge of the French language is too imperfect to enable him to read it correctly. The alternative lies between these suppositions, or a deliberate falsification of the author. *These are not Grisolle's statistics!* They are the results of cases collected from various sources to which Grisolle refers in order to disprove them by his own experience. Grisolle's statistics are as follows: Of 280 cases the pneumonia was double in 17! He devotes more than a page to this topic, so that a careful and competent reader could not overlook it. (*Vide op. cit., page* 30.) He accounts for previous observations showing so large a proportion of cases of double pneumonia on the supposition that capillary bronchitis was frequently mistaken for pneumonic inflammation; and he gives in confirmation of the correctness of his own statistics the results reported by Barth, of cases occurring at the *clinique* of Chomel. Of 125 cases collected by Barth, 8 only were double! On the strength, then, of the very authority which Dr. Burwell cites to convict me of inaccuracy, I reäffirm the statement that "*double pneumonia is exceedingly rare in the adult.*" It is not so rare, however, as *chronic* pneumonia. Grisolle, up to the time his work was written, had met mith one case only of the latter. Dr. Burwell's experience in this particular is also very peculiar. He has reported 4 cases out of 52 (including pneumonia in children) collected in eighteen months!

*Stricture No.* 4. I quote the following from Dr. Burwell's paper: "Another question suggested by the report is, the propriety of calling the consecutive inflammation of the second lung a complication. I fully understand the writer of the report to do this; and I am not clear whether he does not also consider the implication of a second lobe as a complication." Dr. B. complacently devotes nearly a page in combatting a man of straw of his own production. I do not call the consecutive inflammation of the second lung or the implication of a second lobe, complications. It would of course be as absurd to do this as to call an erysipelatous inflammation on one side of the face a complication of that on the other side. Dr. B. cannot understand me in this sense except by a forced construction of language, and I have only to add that, although not so intended, it is a compliment to my report when so zealous a critic is obliged to resort to a stricture of this description.

*Stricture No.* 5. In my report I say that primary idiopathic pneumonia, disconnected from complications, associated morbid conditions and incidental circumstances, intrinsically tends to recovery. Dr. Burwell calls this a "new position." He does me too much credit. I have no claim to the merit of the discovery. What must be the notions of the physicians of the Vienna Hospital on this point when for several years they have treated the disease simply with hygienic measures? But, not to go abroad, were Dr. B. better acquainted than he appears to be with the views of the profession in his own country and his own neighborhood, he would be the last to impute to me originality, for it would then involve the concession that I had done something creditable.

Here ends the series of exceptions which Dr. B. has taken to the "matters of fact" contained in my report. The remainder of his paper is devoted to an attempt to explain away the statistics which in my report I have cited in proof of the intrinsic tendency of pneumonia to recovery. I shall notice this portion of the paper simply to show that it were quite useless, were I

disposed (which I am not) to argue questions of pathology or therapeutic with Dr. Burwell. I give, in my report, the Vienna statistics embracing 750 cases of pneumonia treated with *tisanes* and gum water, containing opium, between the years 1847 and 1850. Of these cases 69 proved fatal, *none of the fatal cases being exempt from complications.* One would think this was pretty conclusive evidence that *uncomplicated* pneumonia tended intrinsically to recovery! But how does Dr. B. dispose of this evidence? He finds it convenient to make a variety of pneumonia which he styles *congestive.* He disclaims an attempt to give a description of the symptoms, *because he has as yet no recorded cases of it!* He apparently proceeds here as he does with respect to a previous "question of fact," viz., that the proportion of fibrin in typhoid pneumonia is less than the normal average: he pre-judges the question, leaving for future accumulation facts in support of the *a priori* conclusion. A very easy method, this, of establishing doctrines in medicine! But doctrines thus made may prove convenient for the sake of argument. Thus Dr. B. finds no difficulty in disposing of the Vienna cases by saying, he believes "that a majority of them were cases of '*congestive pneumonia*,'" and therefore he adds, "they have no great weight with me in deciding upon the question of bloodletting." Could such reasoning as this be seriously answered? It is plainly either above or below logic.*

Again, I cite the statistics of the returns of the British army and navy showing a rate of mortality of from 3 to 4 per cent. These results he receives with more consideration. The English practitioners, he says, resort to free and early bloodletting. It is easy, then, for Dr. Burwell to jump at the conclusion that these cases were bled. Assuming this, and the statistics suit his purpose exactly! nothing could be better! It does not occur to him to mention *congestive pneumonia* in this connection. No. These were all cases of *sthenic pneumonia*, blood was freely drawn, and so "the most inveterate stickler for bleeding could ask nothing better." Is there any reply to this mode of reasoning?

Still, again, Dr. Ames, of Alabama, treated 55 cases without bloodletting, and but a single death occurred. Does this argue aught in favor of the intrinsic tendency of pneumonia to recovery? Not at all in the opinion of Dr. B. Why? Because the cases occurred in a "part of the country where the disease prevails in an epidemic form associated frequently with phenomena distinguished as miasmatic." It does not appear to have occurred to

---

* *Rokitansky and the other distinguished pathologists of Vienna, ought to be informed of this discovery!*

Dr. B. that in an epidemic form and associated with miasmatic phenmonea, the disease is more apt to prove fatal, and that this might furnish an explanation of a large, but certainly not of a small rate of mortality.

Finally, I give the duration of the disease in 6 cases occurring in my own practice during the summer in which the patients were not bled. There is a slight difference only as respects the duration between these, and the cases reported to the association by Dr. B., in which he had practiced bloodletting. How does he attempt to explain away these statistics? Why, *my* cases were mild, and *his* cases were severe, although, as I state distinctly in my report, in one-half of my cases an entire lung was consolidated, while he affirms that in only one of eight cases treated by him during the summer did the inflammation extend over an entire lobe!

I have referred to these points simply by way of an apology (should any be required) to my brethren of the association for declining to engage in any discussion with my colleague on the committee of the subjects assigned to us. If he persuades himself that he has demolished the proof adduced by me of the intrinsic tendency of pneumonia (under the qualifications stated) to recovery, he is fortunate, as it seems to me, in being so easily gratified. I am satisfied to refer others to the facts presented in my report, without farther comment.

Yery respectfully,

Yours,

AUSTIN FLINT.

